# Western Pacific ALS-PDC: a prototypical neurodegenerative disorder linked to DNA damage and aberrant proteogenesis?

**DOI:** 10.3389/fneur.2012.00180

**Published:** 2012-12-21

**Authors:** Peter S. Spencer, Rebecca C. Fry, Valerie S. Palmer, Glen E. Kisby

**Affiliations:** ^1^Global Health Center, Oregon Health and Science UniversityPortland, OR, USA; ^2^Gillings School of Global Public Health, University of North CarolinaChapel Hill, NC, USA; ^3^Basic Medical Sciences, College of Osteopathic Medicine of the Pacific Northwest, Western University of Health SciencesLebanon, OR, USA

**A commentary on**

**Unraveling 50-year-old clues linking neurodegeneration and cancer to cycad toxins: are microRNAs common mediators?**

by Spencer, P., Fry, R. C., and Kisby, G. E. (2012). Front. Gene. 3:192. doi: 10.3389/fgene.2012.00192

The Western Pacific amyotrophic lateral sclerosis and parkinsonism-dementia complex (ALS-PDC) has been described as a Rosetta Stone that bears the essential clue to understanding the etiopathogenesis of related neurodegenerative diseases. The three clinical forms (ALS, atypical parkinsonism with dementia, and dementia alone) have a single pathology (polyproteinopathy, notably tauopathy), just as the Rosetta Stone is inscribed with three distinct scripts bearing a common message. As recently discussed (Kisby and Spencer, 2011), studies of ALS-PDC in the three geographically separate and genetically distinct island populations (Chamorros on Guam; Japanese in Honshu Island's Kii Peninsula; and Papuan New Guineans in Irian Jaya, Indonesia) show:

- ALS-PDC is primarily if not exclusively an environmental disease: no gene mutations identified in related neurodegenerative disorders are found in Guam and Kii-Japan cases, and disease rates have steadily declined in the three affected populations. Emigrants from Guam may develop ALS-PDC years or decades later, but disease risk is absent in their offspring who were born and live abroad. Conversely, Filipino and other immigrants who adopt the Chamorro lifestyle on Guam may acquire the disease.
- As ALS-PDC declined during the twentieth century, the disease changed its clinical face from ALS in the first third of life, to PD in the second, and D in the third, a pattern consistent with a dose-related response to an environmental exposure that waned with modernization. With this hypothesis, those with the highest dose of the putative environmental factor develop fatal ALS (with sub-clinical nigrostriatal damage) relatively shortly after exposure; those with intermediate doses survive with amyotrophy long enough to develop atypical parkinsonism; those with low doses reach old age and display dementia, while others with the lowest exposure have subclinical neurofibrillary disease reminiscent of early aging. Other features of ALS-PDC variably include loss of olfaction, retinal pigment epitheliopathy, and atypical skin cytology.
- The most plausible but unproven trigger decades before the disease surfaces in clinical form is exposure to certain plant-derived neurotoxins in food or medicine, or both. The raw seed of the neurotoxic cycad plant (*Cycas* spp.) was used to heal skin lesions (Guam, Irian Jaya) and as a tonic (Kii). On Guam, processed cycad seed was a Chamorro staple, and the cycad seed-eating flying fox (*Pteropus* sp.) that bioaccumulates the cycad-derived neurotoxin β-*N*-methylamino-L-alanine (L-BMAA) was a delicacy.
- Among the many bioactive chemicals in cycad seed, two with neurotoxic properties are singled out as potential triggers of ALS-PDC: (a) methylazoxymethanol (MAM), a potent genotoxin, carcinogen and developmental neurotoxin that is stored in the plant as an inactive β-glucoside, the concentration of which in cycad flour correlates significantly with incidence rates for ALS and PD in males and females on Guam, unlike the concentration of (b) L-BMAA, a weak excitotoxic amino acid that is taken up by brain tissue and possibly undergoes proteogenesis, resulting in misfolded proteins; daily oral dosing of macaques with L-BMAA for up to 3 months induces a L-dopa-responsive, non-progressive motorsystem disorder with non-excitotoxic cortical and spinal motor neuron pathology. Both L-BMAA and MAM are metabolized to formaldehyde, an established genotoxic agent and human carcinogen.

**Figure 1:**
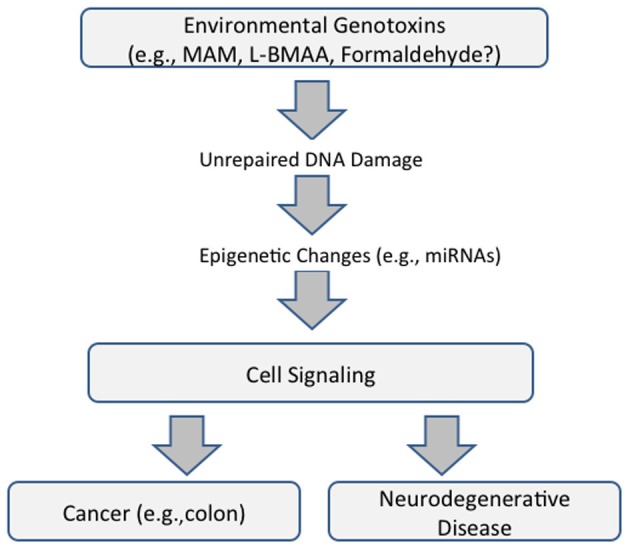
**Proposed common pathway underlying cancer and neurodegenerative disease that is mediated by DNA damage and epigenetic changes (modified from Spencer et al., 2012)**.

Whereas, genotoxin-induced DNA damage is rapidly repaired in non-nervous tissue (i.e., cycling cells), this can persist in the brain because some DNA-repair mechanisms are weakly expressed in post-mitotic cells. MAM-induces *O*^6^-methylguanine (*O*^6^-mG) DNA damage, promutagenic lesions that induce uncontrolled mitoses (tumorigenesis) in mouse epithelial tissues and widespread degeneration in the developing murine brain. *O*^6^-mG lesions are clearly responsible for the MAM-induced neuronal loss because the pathology is greater in mice that lack the DNA-repair enzyme *O*^6^-mG methyltransferase (*Mgmt*^−/−^) and reduced or absent in mice that overexpress MGMT (Kisby and Spencer, 2011). Motor deficits in these mouse mutants are consistent with the extent of DNA damage. In sum, these findings suggest DNA damage is an initial event that leads to brain pathology (Figure 1).

MGMT activity is very low in the young adult human brain and may be absent in mature nerve cells. *Mgmt*^−/−^ mice, an animal model of the young adult human brain, develop persistent brain *O*^6^-mG DNA lesions following a single dose of MAM. In the days following MAM treatment, these DNA lesions modulate key brain cell-signaling pathways that are also perturbed in human neurological disease, notably Alzheimer's disease, Parkinson's disease, and inherited and sporadic forms of ALS. Pathway analysis of MAM-modulated genes that are anchored to DNA damage reveals links with human cancer, genetic disorders, and skin and hair development (Kisby et al., 2011a). Several of these cell-signaling pathways continued to be modulated in the brain of *Mgmt*^−/−^ mice 6 months later, with *de novo* expression changes of numerous genes involving olfaction (Kisby et al., 2011b).

These findings emphasize the relationship between acquired brain tissue DNA damage, modulation of cell-signaling pathways, and the induction of early and persistent molecular changes that lead to neuronal demise. They also reveal important relationships between seemingly disparate diseases—cancer and neurodegeneration—the phenotype of MAM-induced DNA damage being determined, respectively, by the presence or absence of the proliferative capacity of target tissues. The genotoxic properties of MAM and formaldehyde, a common metabolite of MAM and L-BMAA, also involve non-coding RNAs with functional roles in both neurodegeneration and cancer (Spencer et al., 2012) (Figure 1).

While proof is lacking that cycad toxin(s) trigger ALS-PDC, the results of recent biochemical and systems biology studies, coupled with the absence of known mutations in related neurodegenerative disorders, encourage further efforts to examine the molecular and cellular actions of MAM and L-BMAA. These studies not only highlight the response of the brain to unrepaired DNA damage-induced by a genotoxin (e.g., alkylating agent) as a potential initiator of a neurodegenerative process, they also provide a foundation for understanding whether such effects can lead to persistent changes at the protein level (e.g., tau and synuclein), including the erroneous incorporation of foreign amino acids, a subject of recent interest. The ability of MAM to perturb synuclein and several other classes of brain proteins (e.g., calcium homeostasis, mitochondrial and RNA processing) is consistent with this hypothesis (Kisby et al., 2006).
